# Response of Bioactive Metabolite and Biosynthesis Related Genes to Methyl Jasmonate Elicitation in *Codonopsis pilosula*

**DOI:** 10.3390/molecules24030533

**Published:** 2019-02-01

**Authors:** Jiao-jiao Ji, Qi Feng, Hai-feng Sun, Xue-jun Zhang, Xiao-xiao Li, Jian-kuan Li, Jian-ping Gao

**Affiliations:** 1 College of Pharmacy, Shanxi Medical University, Taiyuan 030001, China; jijiao963@163.com (J.J.); 18435147963@163.com (Q.F.); z.xuejun@outlook.com (X.Z.); jijiao963@126.com (X.L.); lijiankuan2004@163.com (J.L.); 2 College of Chemistry & Chemical Engineering, Shanxi University, Taiyuan 030001, China; haifeng@sxu.edu.cn; 3 Dao-di Herbs Resources Development Engineering Research Center of Shanxi Province, Taiyuan 030001, China

**Keywords:** *Codonopsis pilosula*, MeJA, ^1^H-NMR, metabolite, gene expression

## Abstract

Bioactive metabolites in *Codonopsis pilosula* are of particular interest as an immunostimulant. Methyl jasmonate (MeJA) plays an important role in the elicitation of metabolite biosynthesis. Here, we explored the response of metabolites to MeJA elicitation in *C. pilosula* adventitious roots and multiple shoots. The results showed that the biomass, polysaccharide, and lobetyolin content of adventitious roots exhibited the highest increases with 100 µmol·L^−1^ MeJA at the 16th day of subculture, whereas the atractylenolide III (a terpenoid) content increased extremely with 50 µmol·L^−1^ MeJA treatment at the 7th day of subculture. In addition, the biomass and lobetyolin content significantly increased at the 4th day after treatment. Similarly, the polysaccharide and lobetyolin content increased in multiple shoots. Further identification of different metabolites responding to MeJA by ^1^H-NMR showed an extremely significant increase of the lobetyolinin level, which coincided with lobetyolin. Accordingly, the precursor, fatty acids, showed a highly significant decrease in their levels. Furthermore, a significant increase in β-d-fructose-butanol glycoside was detected, which was accompanied by a decrease in the sucrose level. Accordingly, the enzyme genes responsible for terpenoid and carbohydrate biosynthesis, *CpUGPase*, and *CpPMK*, were up regulated. In conclusion, MeJA promoted culture growth and accelerated bioactive metabolite accumulation by regulating the expression of the metabolite biosynthesis related genes, *CpUGPase* and *CpPMK* in *C. pilosula*.

## 1. Introduction

Over the last two decades, the use of herbal medicines has expanded globally, and the medicines have gained considerable attention because of their good therapeutic performance [1]. It has been reported by WHO (world health organization) that 80% of the world’s population rely on medicinal plants for their primary healthcare [2]. Natural products from medical plants include primary and secondary metabolites. In most cases, secondary metabolites that are used as pharmaceuticals are composed of alkaloids, glycosides, flavones, alkynes, and so on, which are desired for their therapeutic values [3,4]. However, the production of secondary metabolites in plants is usually low (less than 1% dry weight) and it greatly depends on the physiological and development stage of the plant [5,6]. 

To overcome this problem, variable in vitro techniques have been widely performed to induce the biosynthesis of bioactive secondary metabolites using biotic or abiotic elicitors [7,8]. Moreover, plant secondary metabolites can also be artificially stimulated by plant hormonal chemicals under natural conditions [9,10]. As one of the representative hormones associated with the plant defense response, MeJA can activate plant defense mechanisms in response to environmental stresses, such as wounding, chilling, UV light, and insect attack, by triggering the biosynthesis of particular enzymes responsible for the production of defensive chemicals and pathogenesis-related (PR) proteins [11,12,13]. The enhanced elicitation of plant secondary metabolites ensures the plant’s survival, persistence, and competitiveness. The application of exogenous MeJA dramatically enhances the content of a few secondary metabolites, including phenols [14], terpenoids [15], anthocyanins [16], polyamines [17], coumarin [18], and alkaloids [19], not only in the plant cell cultures, but also in intact plants [18]. In the root suspension of *Ajuga bracteosa*, MeJA was used as an effective elicitor which induced enhancement in phenolic acid and flavonoid content [20]. 

The dried roots of *Codonopsis pilosula* (Franch.) Nannf. are traditionally used in Chinese medicine for replenishing qi (vital energy) deficiency, strengthening the immune system, improving poor gastrointestinal function, curing gastric ulcers, and appetite [21]. The bioactive constituents are mainly composed of alkaloids [22], phenylpropanoids, polyacetylenes [23], polysaccharides [24], and terpenoids [25]. Lobetyolinin and lobetyolin, which is effective in protecting the gastric mucosa, are the major ingredients of polyacetylenes in *C. pilosula* [26], while the most common terpenoid is atractylenolide III, which has anti-inflammatory activity [27]. In addition, Codonopsis polysaccharides (CPPs) are capable of modulating the immune system functions, preventing tumor growth, improving memory, and increasing hemoglobin [28,29]. However, the elicitation of these metabolites by MeJA has not been reported in *C. pilosula*.

In this study, we focused on investigating the effect and mechanism of MeJA elicitation on the biosynthesis and accumulation of the bioactive metabolites described above in the multiple shoot and adventitious root of *C. pilosula*. Following the optimization of MeJA treatment, dynamics of the bioactive components in the adventitious root and the effect of MeJA on multiple shoots were performed. Thereupon, several different metabolites were identified using ^1^H-NMR analysis in the adventitious roots with MeJA treatment. Finally, the expression pattern of genes involved in metabolite biosynthesis was analyzed to explore the molecular mechanism. The results should provide a theoretical foundation for modulating the accumulation of targeted bioactive ingredients in *C. pilosula*.

## 2. Results

### 2.1. Optimization of the MeJA Treatment

**Figure 1:**
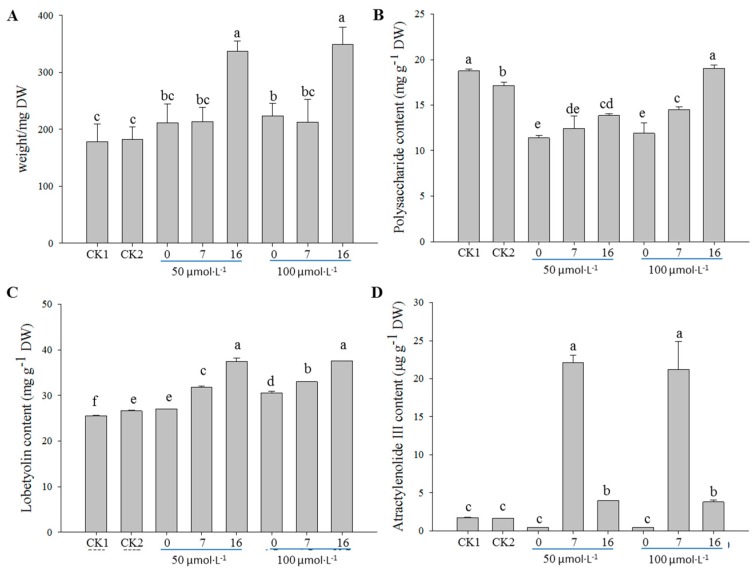
Effect of different time to start treatment (at the 0, 7th, and 16th day of subculture) on biomass (**A**), contents of polysaccharide (**B**), lobetyolin (**C**), and atractylenolide III (**D**) in the adventitious roots of *C. pilosula* treated with 50 and 100 µmol·L^−1^ methyl jasmonate (MeJA), respectively. The samples were collected at the 24th day of subculture and then analyzed. CK1, blank control; CK2, solvent control. Statistically significant differences between the means were determined using Fisher’s LSD test *p* < 0.05) and indicated with different lowercase letters.

Comprehensively considering the medicinal value, three effective ingredients in *C. pilosula*, including polysaccharide, lobetyolin, and atractylenolide III, were used as chemical indexes. Compared with the controls, a noticeable enhancement in the biomass and lobetyolin content was detected when the treatment was performed at the 16th day of subculture, regardless of the concentration of MeJA applied (Figure 1A,C). However, the highest content of atractylenolide III was observed when the treatment was performed at the 7th day of subculture, followed by the treatment at the 16th day (Figure 1D). On the contrary, the polysaccharide content remarkably decreased from the application of MeJA, except for the treatment with 100 µmol·L^−1^ at the 16th day of subculture (Figure 1B).

**Figure 2:**
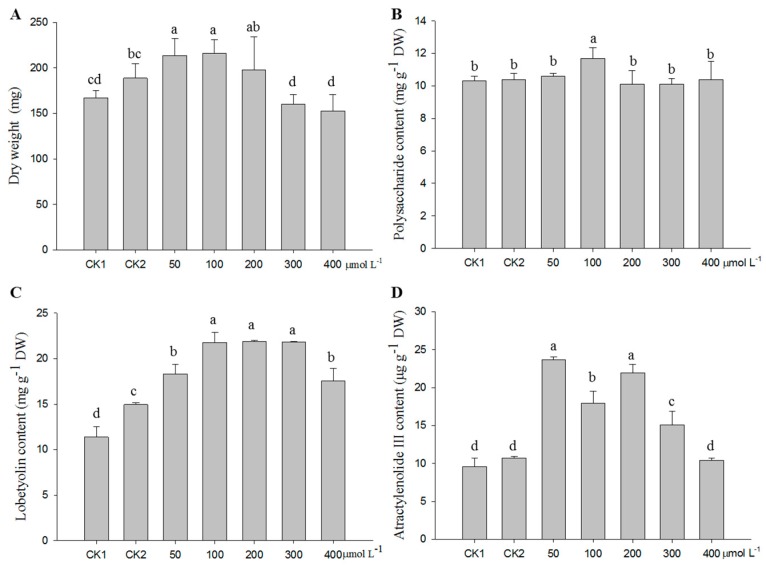
Effect of different concentrations of MeJA on biomass (**A**), contents of polysaccharide (**B**), lobetyolin (**C**), and atractylenolide III (**D**) in the adventitious roots of *C. pilosula*. CK1, blank control; CK2, solvent control. Statistically significant differences between the means were determined using Fisher’s LSD test *p* < 0.05) and indicated with different lowercase letters.

According to the above results, the adventitious roots were treated with MeJA at the 16th day of subculture to analyze the effect of different MeJA concentrations. Results showed that the biomass of the adventitious roots significantly increased with the treatment of 100 µmol·L^−1^ MeJA compared to the controls, whereas a high MeJA concentration (300 and 400 µmol·L^−1^) had an inhibition effect on growth (Figure 2A). Upon the 100 µmol·L^−1^ MeJA treatment, the samples contained a significantly higher polysaccharide content than the controls and the other treatments (Figure 2B). Lobetyolin content in the treatments with 100 µmol·L^−1^ MeJA was significantly higher than the controls and the treatment with 50 µmol·L^−1^ MeJA, with no differences among the treatments with 100, 200, and 300 µmol·L^−1^ MeJA (Figure 2C). For atractylenolide III, all the treatments noticeably promoted its accumulation, excluding the treatment of 400 µmol·L^−1^ MeJA. Relatively, the treatments with 50 and 200 µmol·L^−1^ MeJA had the highest content of atractylenolide III in the adventitious roots (Figure 2D).

### 2.2. Dynamics of Lobetyolin and Atractylenolide III Accumulation under MeJA Treatment

**Figure 3:**
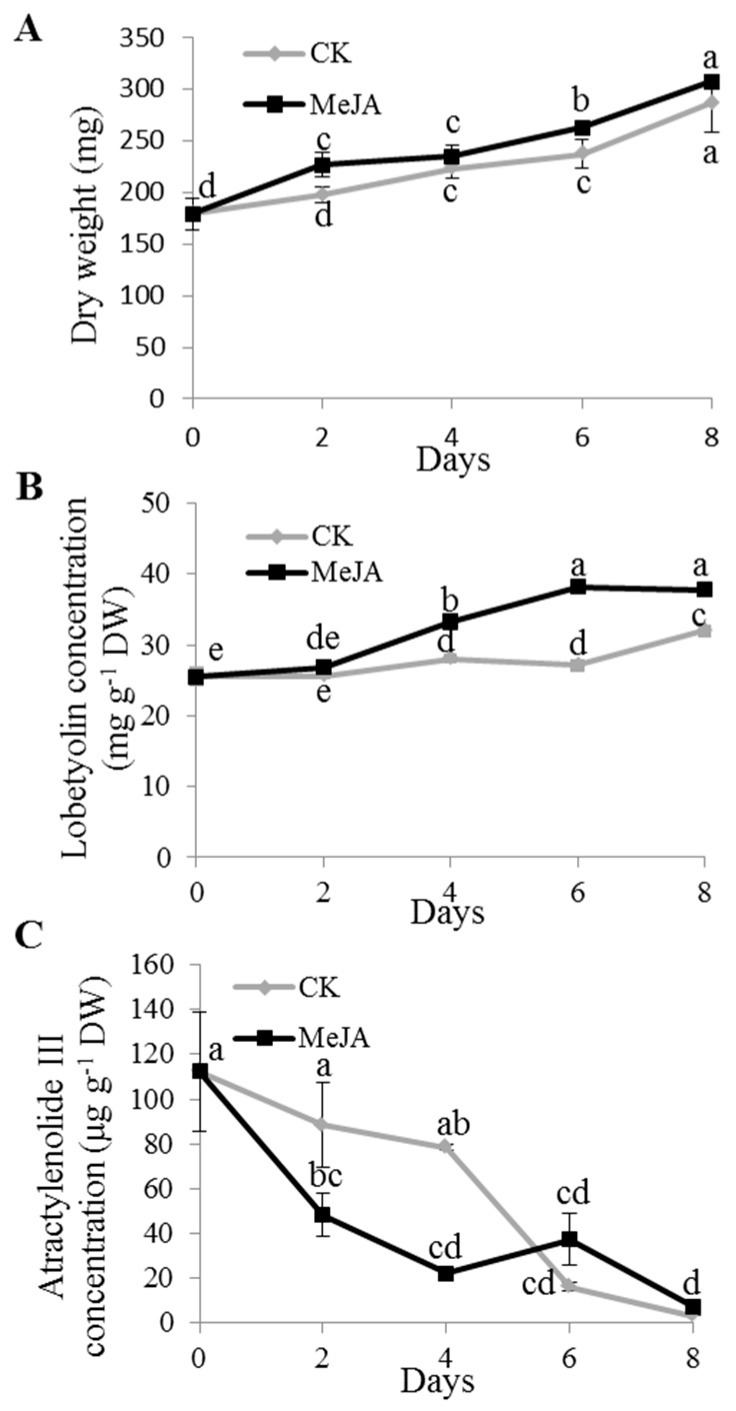
Dynamics of biomass (**A**), accumulation of lobetyolin (**B**), and atractylenolide III (**C**) in the adventitious roots of *C. pilosula* at 0, 2, 4, 6, 8 days after MeJA treatment. CK, blank control. Statistically significant differences between the means were determined using Fisher’s LSD test *p* < 0.05) and indicated with different lowercase letters.

To observe the response time point of metabolite to MeJA, based on the above results, 100 µmol·L^−1^ MeJA was applied to analyze the dynamic of lobetyolin accumulation in the adventitious roots, while the application of 50 µmol·L^−1^ MeJA was performed to dynamically determine the atractylenolide III content. Additionally, the adventitious roots were treated with MeJA at the 16th day of subculture. The dynamics of polysaccharide accumulation were not included for the obscure change upon MeJA treatment in the above studies. The results showed that the biomass gradually increased with time, both in the control and in the treatment. Moreover, the dry weight of the treatment was moderately higher than the control (Figure 3A). The lobetyolin content sharply increased at the 4th day after the treatment in the adventitious roots, while lobetyolin in the control had significant increased at the 8th day after treatment, with no obvious differences within 6 days, but still being lower than that in the treatment (Figure 3B). Overall, the atractylenolide III content decreased during the treatment course, both in the control and the treatment (Figure 3C).

### 2.3. Effect of MeJA on Metabolite Accumulation in C. pilosula Multiple Shoots and Adventitious Roots

**Figure 4:**
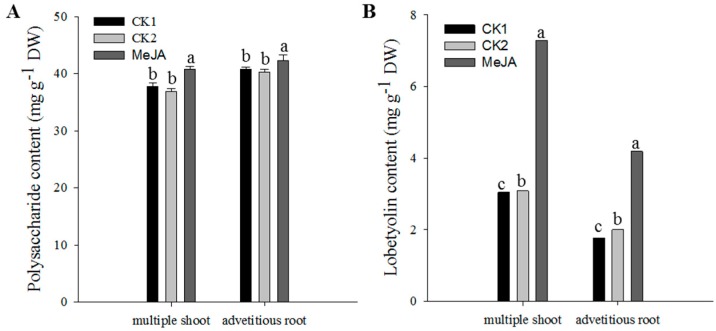
Effect of MeJA on the accumulation of polysaccharide (**A**) and lobetyolin (**B**) in multiple shoots using the adventitious root as the reference. CK1, blank control; CK2, solvent control. Statistically significant differences between the means were determined using Fisher’s LSD test *p* < 0.05) and indicated with different lowercase letters.

Since MeJA treatment could enhance the bioactive metabolite accumulation in the adventitious roots, it was plausible that MeJA might exert a similar effect on the multiple shoots. After treatment with 100 µmol·L^−1^ MeJA at the 16th day of subculture, the multiple shoots presented significantly higher levels of polysaccharide (Figure 4A) and lobetyolin (Figure 4B), especially the latter, on average 2.4-times, which corresponded with the results found in the adventitious roots. The atractylenolide III was not detected in the multiple shoots treated with 100 µmol·L^−1^ MeJA, which might have been caused by the application of an unsuitable MeJA concentration or an excessively low content in the sample that could not be detected by HPLC (high performance liquid chromatography).

### 2.4. Metabolomic Profiling of C. pilosula Adventitious Root by ^1^H-NMR

As mentioned above, the MeJA treatment could enhance the accumulations of polysaccharide, lobetyolin, and atractylenolide III. To identify the metabolites involved in the induction of MeJA in *C. pilosula*, metabolomic profiling was performed by ^1^H-NMR using the control and treated adventitious root with 100 µmol·L^−1^ MeJA at the 16th day of subculture.

**Figure 5:**
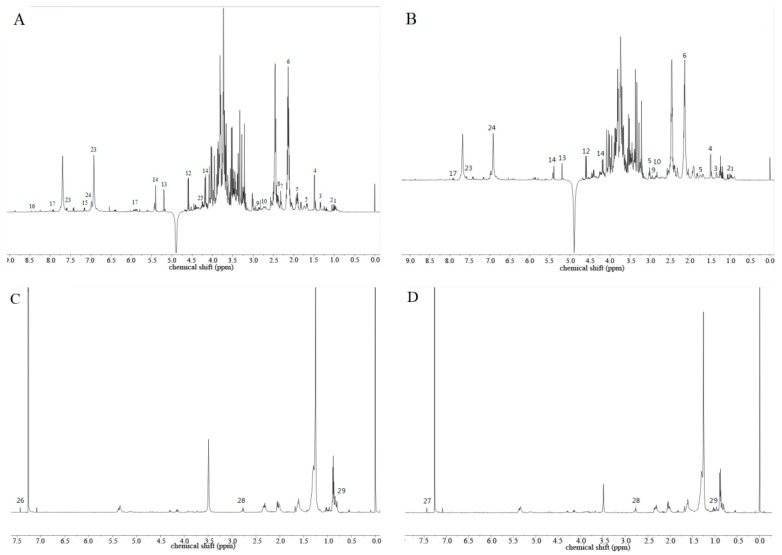
Spectra of ^1^H-NMR of the adventitious root treated with 100 µmol·L^−1^ MeJA at the 16th day of subculture. (**A**) Methanol aqueous phase of control. (**B**) Methanol aqueous phase of treatment. (**C**) Chloroform phase of control. (**D**) Chloroform phase of treatment. The compounds represented by numbers were listed in Table 1.

Based on the known chemical structures and spectra, a total of 31 metabolites were identified based on the ^1^H-NMR spectrums (Figure 5). In the methanol aqueous phase, ingredients with a high polarity shared the largest portion. The spectrum could be roughly divided into three regions. The high field region (δ 3.10~0.00) mainly contained organic acid and amino acid. The carbohydrate region (δ 5.50~3.10) mainly contained α-glucose, β-glucose, sucrose, and phenylpropanoids (e.g., tangshenoside I and IV). The low field region (δ 10.4~5.50) mainly contained polyphenols, such as gallic acid (Figure 5A,C). The extracts in the chloroform phase mainly constituted the ingredients with low polarity, such as fatty acids and β-sitosterol (Figure 5C,D). The identified compounds were listed in Table 1.

### 2.5. Identification of the Differential Metabolites in C. pilosula Adventitious Roots upon MeJA Treatment

As depicted in the score scatter plot of the PLS-DA (Figure S1A,C), the treatment could be thoroughly separated from the control along the *t* [1] axis. The permutation experiment, verifying the validity of the PLS-DA model, revealed that the intersection of the blue regression line and the vertical axis was below zero, and any R^2^ and Q^2^ values on the left, that were produced by the random arrangement, were less than that on the right. Meanwhile, the two regression line slope was larger, and the difference value between the two values on the right end was smaller (Figure S1B,D). All these results indicated that the predictive ability of the archetype was larger than that of the randomly arranged *y* variable, which proved that the model was effective to different metabolites induced by the MeJA treatment.

**Figure 6:**
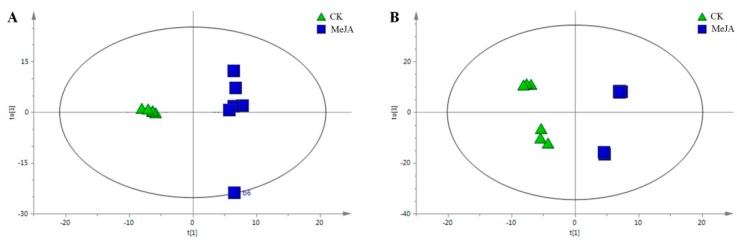
Orthogonal partial least square-discriminant analysis (OPLS-DA) scores scatter plot showing the complete separation of the metabolite profiles of the adventitious root grown under normal (CK) and MeJA elicitation conditions. (**A**) Methanol aqueous phase. (**B**) Chloroform phase.

Subsequently, the OPLS-DA analysis showed that the metabolic profiling of the two groups showed a clear segregation along the *t* [1] axis. The separation of the two groups in the OPLS-DA plot indicated that some chemical components between the two groups varied and it further suggested the existence of one or more chemical biomarkers. Moreover, the different individuals between the groups showed an obvious aggregation (Figure 6). All these results indicated that the MeJA treatment had an apparent effect on the metabolite accumulation in the *C. pilosula* adventitious root.

**Figure 7:**
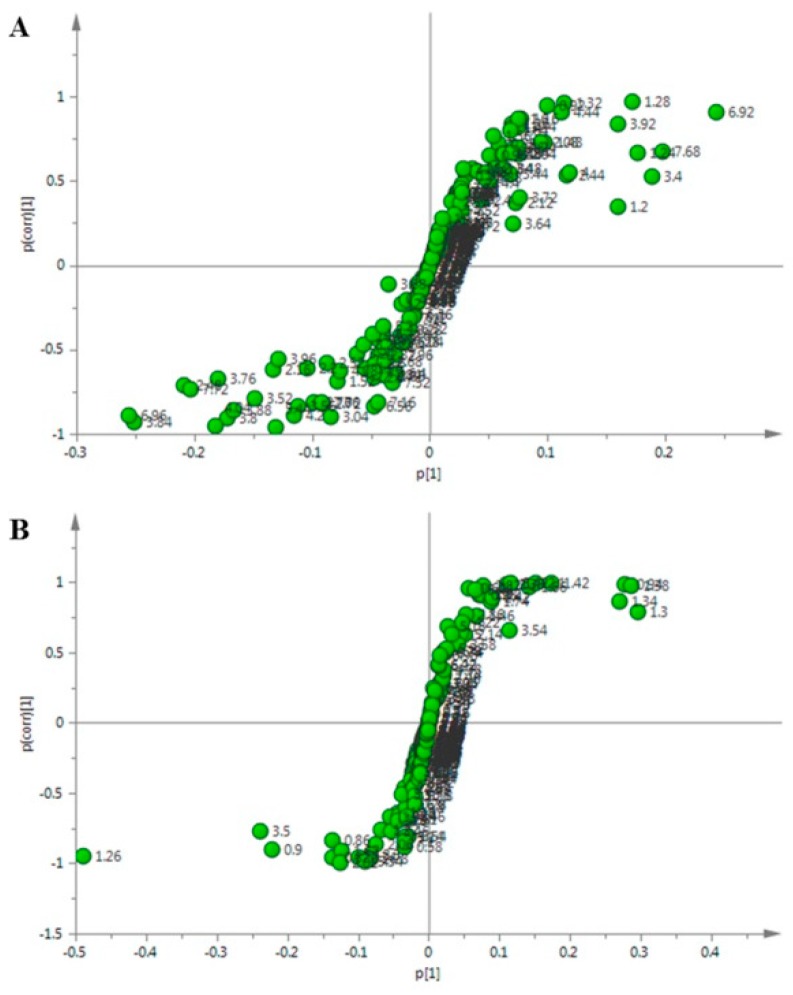
S-plot analysis showing the potential biomarkers in the adventitious root after the MeJA treatment. (**A**) Methanol aqueous phase. (**B**) Chloroform phase.

Finally, the S-plot analysis was performed to identify the putative biomarkers. The dots farther from the origin made a greater contribution to the differences between the groups, which could be considered as the potential biomarker (Figure 7).

**Figure 8:**
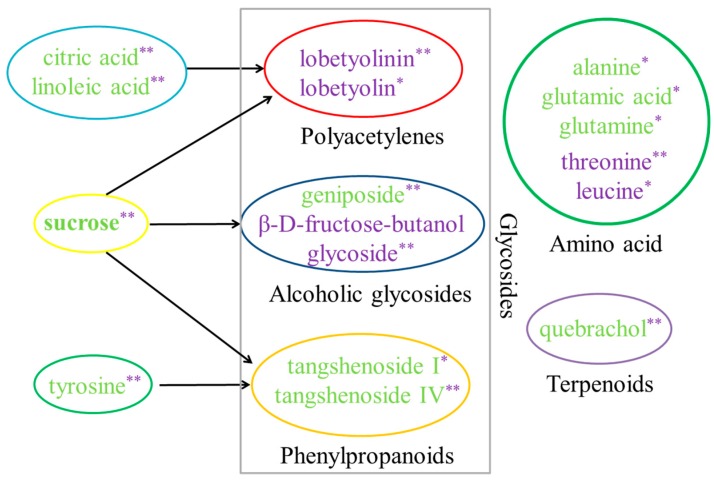
Biomarkers identified in the MeJA treated adventitious root of *C. pilosula*. The differential analysis of the compound level was analyzed using the *t*-test. Purple font indicated up regulation. Green font indicated down regulation. Black arrow indicated the relationship between the precursor and metabolite. Aqua circle, fatty acid; Red circle, polyacetylenes; Green circle, amino acids; Blue circle, glycosides; Yellow circle, carbohydrates; Orange circle, phenylpropanoids; Purple circle, terpenoids. * indicated *p* < 0.05. ** indicated *p* < 0.01.

In total, eighteen compounds were identified and classified into or involved in the biosynthesis of amino acid, glycosides, sugar, and terpene, respectively (Figure 8). The *t*-test showed that the level of threonine and leucine significantly increased among the six amino acids, while the others (i.e., tyrosine, alanine, glutamic acid, and glutamine) decreased. Amongst the 18 biomarkers, the glycosides shared the larger part, including polyacetylene, alcoholic glycoside, and phenylpropanoids (Figure 8). Lobetyolinin and lobetyolin, the two polyacetylenes, exhibited a significantly increased level upon treatment with the MeJA, especially lobetyolinin. Meanwhile, the precursors of polyacetylene biosynthesis, i.e., citric acid and linoleic acid [39], exhibited extremely significant decreases in their levels. In addition, the level of β-d-fructose-butanol glycoside, an alcoholic glycoside, was most elevated after the MeJA treatment, accompanied by an extreme decrease in the level of sucrose. Moreover, another alcoholic glycoside, geniposide, exhibited a decreased change. Meanwhile, the MeJA led to a significant decrease of the two lignanoids, tangshenoside I and IV, while similar changes were found in the level of their potential precursors, tyrosine and sucrose. Compared with the control, the level of terpene and quebrachol, also decreased remarkably in the treatment with MeJA.

### 2.6. Effect of MeJA on the Expression of the Genes Involved in the Biosynthesis of the Main Bioactive Components in C. pilosula

**Figure 9:**
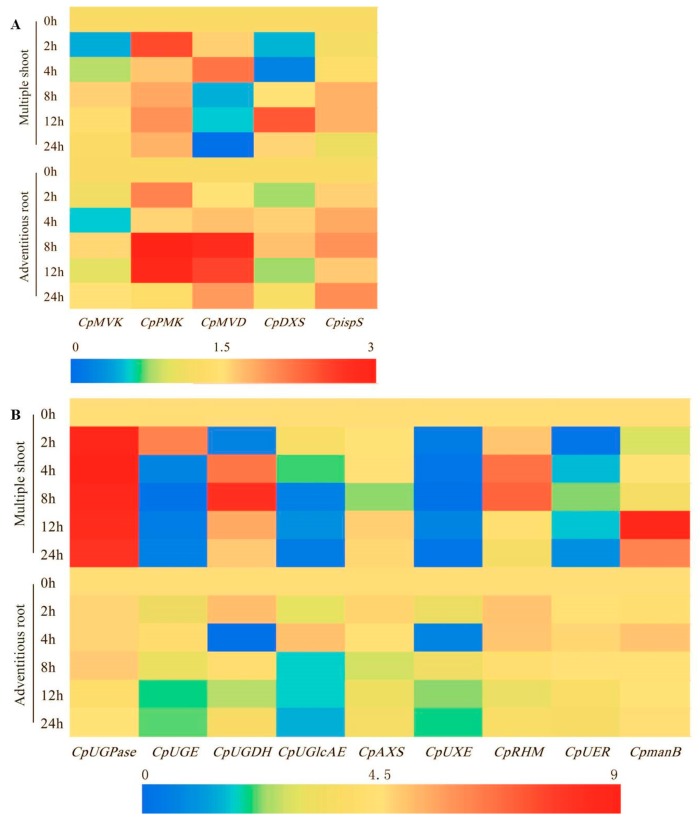
Expression patterns of the genes involved in the biosynthesis of terpenes (**A**), polysaccharides and glycosides (**B**) in *C. pilosula* adventitious root and multiple shoots with MeJA treatment. *CpGAPDH* was used as the reference gene. Blue indicated the lowest expression. Red indicated the highest expression.

To gain insight into the molecular mechanism of the enhanced accumulation of bioactive compounds induced by MeJA elicitation, we analyzed the expression patterns of the genes associated with the biosynthesis of polysaccharide, glycosides, and atractylenolide III (belonging to terpenoid). Overall, the expression of *CpPMK* and *CpispS* genes, which participated in terpenoid synthesis, was up regulated after elicitation both in the multiple shoots and the adventitious roots. In contrast, the *CpMVD* gene in the multiple shoots showed down regulation, which was contrary to that in the adventitious roots after a 4 h MeJA treatment (Figure 9A). Remarkably, the expression of *CpUGPase*, which was involved in the carbohydrate synthesis, sharply increased after MeJA elicitation in the multiple shoots, whilst in the adventitious roots, it had no obvious changes. On the other hand, the *CpUGE*, *CpUGDH*, *CpUGlcAE*, *CpUXE*, *CpUER* genes were down regulated in both the shoots and the roots on the whole. However, *CpmanB* gene expression increased and reached the peak at the 12-h time point in the multiple shoots (Figure 9B).

## 3. Discussion

As an elicitor, MeJA is not only the main signal molecule for secondary metabolite production [40], but it also functions in regulating plant growth at different scales (i.e., from cell division to organ development) [13]. There is always a balance between growth and defense. Our studies showed that the biomass of the adventitious roots increased with the MeJA treatment (Figure 1, Figure 2, and Figure 3A). A similar result was observed in the leaves of *Matricaria chamomilla* after foliar application of 400 µmol·L^−1^ MeJA [18]. It might have been because the adventitious roots were affected not only at the cell expansion level, but also at the cell proliferation level [13]. Thus far, a growing body of publications has shown that the addition of different concentrations of MeJA to the medium exhibits positive roles on plant growth. However, high MeJA concentrations inhibited cell growth, which might have been caused by inhibitory photosynthesis, especially on the biosynthesis of photosynthetic pigments [18]. These coincident results were found not only in our study (Figure 2A), but also in *Chlorella vulgaris* with the treatment of higher concentrations of MeJA (10–100 µmol·L^−1^) [41].

Elicitation of metabolite and related gene expression by MeJA has also been reported in many plants [42,43]. Using ^1^H-NMR analysis, 18 different metabolites were observed, including amino acids, terpenoids, carbohydrates, and glycosides in *C. pilosula* adventitious roots with MeJA treatment. In general, polysaccharide ingredients are known for their activity in strengthening the human body’s immunity, i.e., ganoderma polysaccharides and ginseng polysaccharides. In addition, codonopsis polysaccharides (CPPs) are capable of preventing tumor growth, improving memory, curing diabetes, and increasing hemoglobin [24]. Yang et al. [44] demonstrated that CPPs are an acidic hetero-polysaccharide composed of arabinose, glucose, rhamnose, galactose, mannose, glucuronic acid, and galacturonic acid. In a previous study, we speculated that sucrose should be used as the primary starting material for CPPs biosynthesis using transcriptome sequencing and gene function annotation [24]. In this study, the sucrose level was lower in the adventitious roots treated with 100 µmol·L^−1^ MeJA (Figure 8), coinciding with significantly increased polysaccharide content (Figure 2B), supporting the above speculation. In *Astragalus membranaceus*, the activity of the UGPase enzyme, catalyzing the reversible reaction between glucose 1-phosphate and UDP-glucose, was positively correlated with the polysaccharide content [45]. Elicitation of transcripts by MeJA in *Lycoris aurea* has also been reported [42]. In our work, the transcriptional abundance of *CpUGPase* sharply increased in the adventitious roots upon treatment with MeJA (Figure 9B), which was consistent with increased polysaccharides. In *Populus*, the overexpression of *UGPase* reduced the sugar and starch levels and increased the phenolics, such as caffeoyl and feruloyl conjugates [46]. Likewise, a significant decrease of sucrose level was observed in this study, while glycosides, including β-d-fructose-butanol glycoside, lobetyolinin, and lobetyolin exhibited a significant increase in their levels. The three glycosides in *C*. *pilosula* contained a fructosyl and a glycosyl, respectively. On the other hand, sucrose synthase converts sucrose to UDP-glucose and fructose [24]. Therefore, we speculated that the decreased sucrose level and increased expression of *CpUGPase* might result in the increased level of UDP-glucose, glucose 1-phosphate, and fructose, which could provide glycosyl precursor for the biosynthesis of polysaccharide and glycosides, such as β-d-fructose-butanol glycoside, lobetyolinin, and lobetyolin. Further works should be carried out to verify this speculation. However, geniposide, and tangshenoside I and IV, showed similar decreases in their levels to the precursors, sucrose and tyrosine. It might have been caused by the diversity of biosynthesis of the compounds in the plants.

CpUGE, CpUGDH, CpUGlcAE, CpUXE, and CpUER take part in the biosynthesis of galactose, arabinose, xylose, and rhamnose, which are the precursors of heteropolysaccharide [24]. Our previous study found that the polysaccharide in Condonopsis Radix was mainly fructan [29]. Down-regulation of the *CpUGE*, *CpUGDH*, *CpUGlcAE*, *CpUXE*, *CpUER* genes might indicate that the increase in the polysaccharide content might result mainly from the increase in the fructan content in the adventitious root. This speculation could be proved by the up-regulation of *CpmanB,* which was involved in the fructose biosynthesis.

Lobetyolinin and lobetyolin, two main polyacetylene compounds in *C. pilosula*, were composed of a fatty acid chain and a glucosyl [47]. Previous studies showed that pulsed light could enhance the level of polyacetylene in carrots [48]. However, there are limited reports on the effect of MeJA on polyacetylene biosynthesis. In this study, significantly increased levels of lobetyolinin and lobetyolin were observed in the adventitious roots and multiple shoots of *C. pilosula* after MeJA treatment. In the pathway of polyacetylenes biosynthesis, oleic acid is catalyzed to produce crepenynic acid, then the latter goes through a series of dehydrogenation reactions to form the fatty acid chain [49]. Citric acid and linoleic acid are both closely related to the metabolism of oleic acid. ^1^H-NMR analysis exhibited that fatty acids were negatively correlated with lobetyolin and lobetyolinin levels. These results supported the view that fatty acids participated in the biosynthesis of lobetyolin and lobetyolinin.

Artemisinin, belonging to terpenoids, is one major metabolite with anti-malarial activity. The application of MeJA and jasmonic acid (JA) together with a phytohormone gibberellic acid (GA), increases the accumulation of artemisinin in the cell suspension culture of *Artemisia absinthium* [50]. In *C*. *pilosula*, the main bioactive terpenoid is atractylenolide III. In this study, the enhancement of the atractylenolide III content was observed in the treated adventitious roots and the multiple shoots of *C. pilosula,* treated with MeJA (Figure 1D and Figure 2D). PMK is the crucial enzyme in the mevalonic acid (MVA) pathway, which participated in terpenoid biosynthesis [51]. Here, increased gene expression of *CpPMK* was detected after the MeJA treatment in the multiple shoots and adventitious roots. Hence, we speculated that *CpPMK* might modulate the biosynthesis of atractylenolide III in *C*. *pilosula* by MeJA elicitation. CpMVK, CpMVD, CpDXS, and CpispS are enzymes belonging to the mevalonate pathway, which is one of the branches of terpenoid biosynthesis [52]. Less obvious changes were found in the expression of the *CpMVK, CpDXS*, and *CpispS* genes at 24 h after treatment in the adventitious roots and multiple shoots, which might indicate that these genes did not respond to MeJA, as well as *CpAXS* and *CpRHM* involved in the carbohydrate biosynthesis. *CpMVD* gene expression was up-regulated in the *C. pilosula* adventitious root, which was similar to that in *Ganoderma lucidum* [53]. However, *CpMVD* gene expression was up-regulated and then down-regulated at 4 h after the MeJA treatment in the multiple shoots. It might have been caused by earlier response to the MeJA in multiple shoots, compared with that in the adventitious roots.

## 4. Materials and Methods

### 4.1. Plant Material and Treatment

*C. pilosula* seeds were collected from a field located in Lingchuan, Shanxi province, China. The seeds were treated with warm water for 30 min, then with 400 mg·L^−1^ GA_3_ for 8 h at room temperature. Then the seeds were surface sterilized with 75% alcohol (*v/v*) for 10 s, then deeply sterilized with 1% NaClO for 10 min, and transferred on half-strength Murashige and Skoog (1/2 MS) solid medium (pH 5.8) in the dark at 25 °C until germinated. Afterwards, the germinated seeds were cultured in a growth chamber under 12 h light (25 °C)/12 h dark (20 °C) for 30 d. The caulicle and root of the aseptic seedlings were cut into 1 cm pieces for the culture of the multiple shoots in MS solid medium and the adventitious roots in 1/2 MS solid medium, respectively [54]. The induced adventitious roots with a length of 1 cm were transferred into a 1/2 MS liquid medium for subculture. The induced multiple shoots cultured for 15 days were used for the subculture in MS solid medium.

In order to select a time point for the MeJA treatment, the adventitious roots were treated at the 0, 7th, and 16th day of subculture, with 50 and 100 μmol·L^−1^ MeJA, respectively. At the 24th day of subculture, the root samples were harvested. Based on the selected time, the effects of different concentrations of MeJA (50, 100, 200, 300, and 400 µmol·L^−1^) on adventitious root growth and bioactive compound accumulation, were investigated to select the optimum concentration of MeJA. At the 24th day of subculture, the root samples were harvested. The selected time and concentration were used for the following study.

For the dynamic analysis of bioactive metabolites, the adventitious roots were harvested at 0, 2, 4, 6, 8 d after the MeJA treatment. In addition, the effect of the MeJA on the bioactive metabolite biosynthesis and accumulation in the multiple shoots was analyzed upon the optimum treatment conditions.

The samples were dried at 50 °C to a constant weight, then weighed up for the biomass and chemical analysis.

To analyze the expression of genes involved in the biosynthesis of bioactive metabolites, the multiple shoots and the adventitious roots were harvested at 0, 2, 4, 8, 12, 24 h, respectively, after treatment with 100 µmol·L^−1^ MeJA, then they were immediately frozen in liquid nitrogen and stored at −80 °C.

Samples cultured in normal medium were used as the blank control (CK1) and those in the MeJA-free solution (2.5% alcohol) were used as the solvent control (CK2). All the experiments were replicated three times. Three to five replicas were performed in each replicated experiment.

Upon the optimum MeJA treatment conditions, the adventitious roots were treated and collected at the 24th day of subculture to perform the ^1^H-NMR analysis.

### 4.2. Quantification of Bioactive Metabolites

The content of polysaccharide, lobetyolin, and atractylenolide III was measured using methods described previously in References [55,56].

### 4.3. ^1^H-NMR Analysis

The mixture consisting of 0.2 g dry powder of the adventitious roots, 1.5 mL H_2_O, 1.5 mL methanol, and 3.0 mL chloroform, was ultrasonically treated for 25 min. The methanol and chloroform phase was separated by centrifugation at 3000 rpm for 25 min. After rotary evaporation, the chloroform phase was redissolved in 600 µL chloroform then transferred into the NMR tube. The methanol phase was redissolved in 400 µL deuterated methanol. After centrifugation at 13,000 rpm for 10 min, the supernatant was transferred into the NMR tube. ^1^H-NMR spectra was recorded at 25 °C on a 600 MHz Bruker AVANCE III-600 spectrometer (Bruker, Switzerland), operating at a proton NMR frequency of 600.13 MHz. For analysis of the extracts from the methanol aqueous phase, the noesypprld sequence was adopted using TSP (sodium trimethylsilane propionate) as the internal index and methanol-*d4* as the internal lock, while for the chloroform phase extracts, the zg30 sequence was used with TMS as the interior label and CDCl_3_ as the internal lock. The ^1^H-NMR spectra were calibrated using MestReNova. Spectral intensities were scaled to TMS and reduced to integrated regions of equal width (0.04 ppm), corresponding to the region of δ 0.4 to 10.4. The data was processed using the normalization method. Afterward, partial least square-discriminant analysis (PLS-DA) and orthogonal partial least square-discriminant analysis (OPLS-DA) were performed to identify the differential metabolites using SIMCA-P 13.0 (Umetrics, Umea, Sweden).

### 4.4. Gene Expression Analysis

RNA extraction, cDNA preparation, and qRT-PCR were performed as described by Cao et al. [57]. Gene-specific primers were designed using Primer 5.0 (Primer Premier, Quebec, Canada). Primer specificity was confirmed by blasting each primer sequence against Phytozome2 using the BLASTN algorithm. *GAPDH* was amplified as the reference gene [57]. Details on the specific primers are listed in Table 2.

### 4.5. Statistical Analyses

Values were expressed as the mean ± SE. The data was analyzed using analysis of variance (SAS 8.2, North Carolina State University, Raleigh, NC, USA).

For THE ^1^H-NMR analysis, PLS-DA, OPLS-DA, S-plot, *t*-test were performed. To deconvolute the metabolic alteration caused by the MeJA treatment, a supervised multivariate data analysis, PLS-DA in which two classes (control and MeJA treatment) were used for the Y-matrix combined with X-matrix of NMR data, was applied to the NMR data set. OPLS-DA analysis, which could enable the differences between the two groups reach the maximum, was performed to determine the chemical differential ingredients between the two groups. To further analyze the comparative metabolomics profiles of the treatment and the control, the S-plot analysis and *t*-test were performed as a multivariate analytical technique, a useful tool for identifying putative biomarkers. The mean separation was analyzed using the Fishers LSD test. A *p*-value < 0.05 was considered significant.

## 5. Conclusions

In summary, our work provided evidence that the MeJA treatment could enhance the accumulation of bioactive metabolites in *C. pilosula*, especially polysaccharide, β-d-fructose-butanol glycoside, lobetyolin, and atractylenolide III. The enhancement was coincident with the transcripts of *CpUGPase* and *CpPMK* genes, which participated in the biosynthesis of carbohydrates, glucosides, and terpenoids. Furthermore, the biomass of the adventitious roots and the multiple shoots was improved by the application of a moderate MeJA. In future, discovery and the functional identification of the genes associated with the biosynthesis of the three ingredients will reveal the molecular mechanism of the response of secondary metabolites to MeJA in *C. pilosula*.

## Figures and Tables

**Table 1:** Characteristic chemical shifts (δ in ppm) and coupling constants (*J*) of the compounds detected in the ^1^H-NMR of the control- and MeJA-treated adventitious root of *C. pilosula*.

Peak Number	Compound	δ_H_ (*J* in Hz)	Reference
1	leucine	δ 0.94 (t, 3.3)	[30]
2	valine	δ 1.06 (d, 7.0), 1.01 (d, 7.0)	[30]
3	threonine	δ 1.34 (d, 6.5)	[30]
4	alanine	δ 3.73(m), 1.49 (d, 7.2)	[30]
5	arginine	δ 1.92–1.89 (m), 1.70–1.65 (m)	[30]
6	glutamic acid	δ 2.38(m), 2.13 (m)	[30]
7	acetylglutamate	δ 2.32 (t, 7.2)	[30]
8	glutamine	δ 2.46 (m), 2.13 (m)	[30]
9	asparaginic acid	δ 2.84 (dd, 16.4, 6.5)	[31]
10	citric acid	δ 2.72 (d, 4.6)	[31]
11	choline	δ 3.22 (s)	[31]
12	β-glucose	δ 4.60 (d, 7.9)	[31]
13	α-glucose	δ 5.20 (d, 3.7)	[31]
14	sucrose	δ 5.41 (d, 3.8), 4.18 (d, 8.7)	[31]
15	tyrosine	δ 7.16 (d, 8.7)	[30]
16	formic acid	δ 8.47 (s)	[31]
17	uridine	δ 7.86 (d, 8.7), 5.89 (dd, 10.6, 4.3), 4.35 (d, 7.7), 4.14 (dd, 8.8, 6.2), 3.80 (dd, 6.3, 2.3)	[32]
18	tangshenoside I	δ 4.60 (d, 7.9), 3.75 (s), 2.82 (s), 2.46 (d, 7.5), 1.41 (s)	[33]
19	Tangshenoside IV	δ 6.93 (s), 3.83 (s), 3.80 (s), 3.72 (s), 2.62 (d, 3.2)	[34]
20	lobetyolinin	δ 4.43 (d, 7.3), 4.37 (d, 6.4), 3.74 (dd, 11.5, 5.2), 3.53 (t, 4.3)	[35]
21	lobetyolin	δ 4.35 (d, 7.7), 4.24 (d, 2.6), 4.18 (dd, 9.6, 3.8)	[35]
22	β-d-fructose-butanol glycoside	δ 4.06 (d, 2.5), 1.57–1.51 (m)	[32]
23	vanillic acid	δ 7.54 (d, 2.4), 6.85 (d, 8.0)	[36]
24	aurantiamarin I	δ 6.93 (s), 3.81 (s), 1.06 (d, 7.0)	[35]
25	geniposide	δ 7.51 (s), 5.80 (s),5.17 (d, 3.7), 4.20 (d, 3.3), 3.87 (d, 2.3)	[32]
28	augelicin	δ 6.37 (d, 7.1)	[37]
29	psoralen	δ 7.48 (s), 6.37 (d, 7.1)	[37]
30	linoleic fatty acid	δ 2.77 (t, 6.6), 1.30–1.20 (m)	[38]
31	β-quebrachol	δ 0.89 (t, 5.9), 0.87 (d, 7.0)	[35]

s singlet, d doublet, t triplet, m multiplet.

**Table 2:** Primer sequence used in this study.

Gene	Protein	Primer (5′–3′)	Product (bp)
*CpGAPDH*	glyceraldehyde-3-phosphate dehydrogenase	F: TGCTTCGTTCAACATCATTC	164
		R: CATAACTGGCTGCCTTCTCC	
*CpMVK*	mevalonate kinase	F: GACACAAAAGTTGGGAGGAACAC	126
		R: GGTAGCCAGTTCATTGCTGATAGA	
*CpPMK*	phosphomevalonate kinase	F: CTGCCGTAGTTGCTGCTTTACTT	88
		R: TTCGTGGCTGTTTCTTGGTG	
*CpMVD*	Methylovalerate decarboxylase	F: CAAGATGCTGGCGTTCAGG	92
		R: CCTTTGGTTTTCTGCGTTGG	
*CpDXS*	1-deoxyxylose-5-phosphate synthase	F: TTGGCATAGCCGAACAACA	94
		R: TGGAGGAAAGACGAGTAAATAGCAC	
*CpispS*	isoprene synthase	F: ACGAACACTGCATCAAAGAATCTC	112
		R: TGAACCCCAAAACCATCTCC	
*CpUGPase*	UDP-glucose pyrophosphorylase	F: TTTACCCTTGAGAACGACG	192
		R: TCTGATGGCTATGTGACCC	
*CpUGE*	UDP-glucose isomerase	F: CGGGGTACATCTGTGCTTG	161
		R: ATGCCATACTTTGCCTTCC	
*CpUGDH*	UDP-glucose dehydrogenase	F: GATGCTTATGAGGCGACGAA	193
		R: GAGGCTTACCAATGGAGTAGACAAT	
*CpUGlcAE*	UDP galaotcse 4-epimerase	F: CACGGGATTTTACCTACAT	95
		R: CTTCTTCTTACCGCCTGAT	
*CpAXS*	UDP-apiose/xylose synthase	F: GATAAAGGCGATGACGATA	146
		R: AAGACGGTTCAAGAAGGTG	
*CpUXE*	UDP xylose epiisomerase	F: TGTTGGCACAGGAAGAGGT	97
		R: CCGACGAGGAAGGAAATCA	
*CpRHM*	rhamnose synthase	F: TCGGCATTCGGACTCTAAG	111
		R: TCCTGAAGGTTCTGGCATT	
*CpUER*	UDP-4-keto-6-deoxyglucose isoreductase	F: CCAATCCCCGTAACTTCAT	131
		R: TATTCCAGTCAGGTTCCTCTTT	
*CpmanB*	mannase B	F: AACGCCAACTGAGACAAC	86
		R: GCACTCTTACAGCACCGA	

## Supplementary Materials

The Figure S1 is available online.

## Abbreviations

MeJA	Methyl jasmonate
PLS-DA	Partial least square- discriminant analysis
OPLS-DA	Orthogonal partial least square-discriminant analysis
UGPase	UDP-glucose pyrophosphorylase
PMK	Mevalonate-5-phosphate kinase
