# Supplementary figures and images for: Response of Bioactive Metabolite and Biosynthesis Related Genes to Methyl Jasmonate Elicitation in Codonopsis pilosula
Source: Molecules. 2019 Feb 1;24(3):533. doi: 10.3390/molecules24030533 (PMC6385095; doi:10.3390/molecules24030533)

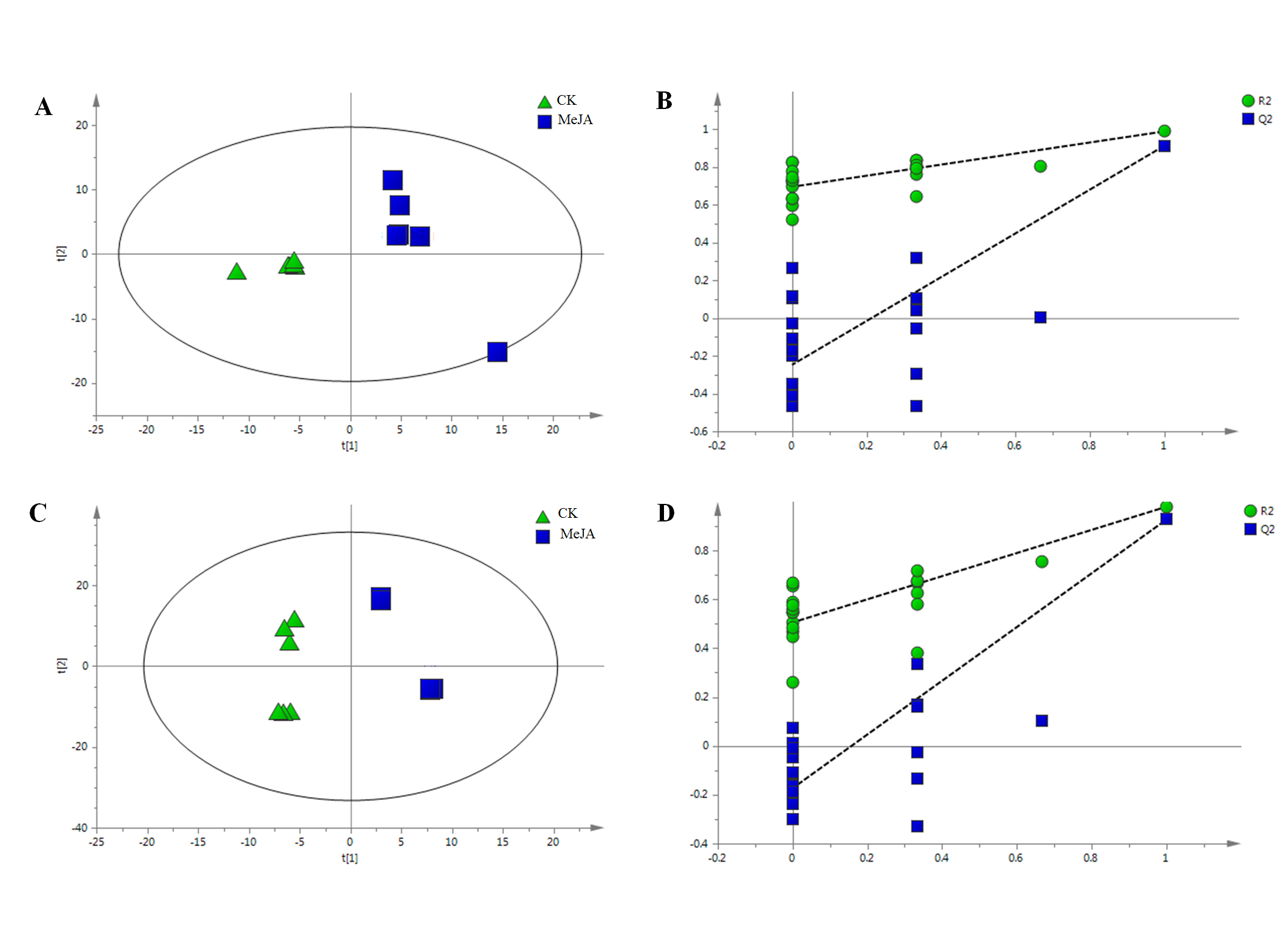

Supplement: Supplementary file 1 [file molecules-24-00533-s001.jpg]
